# Automated Intracranial Hemorrhage Segmentation in CT Images Using Attention U-Net with Deep Supervision and Test-Time Augmentation

**DOI:** 10.3390/diagnostics16213545

**Published:** 2026-10-28

**Authors:** Kenza Chenni, Altin Kuqo, Ergys Xhukellari, Lavdie Rada

**Affiliations:** 1 LIS Laboratory, Department of Electronics, Faculty of Technology, Ferhat Abbas Setif 1 University, Setif 19000, Algeria; kenza.chenni@univ-setif.dz; 2 Neurology Service, Mother Teresa University Hospital Center, 1001 Tirana, Albania; altin.kuqo@umed.edu.al (A.K.); ergysxhukellari.2@gmail.com (E.X.); 3 Faculty of Engineering and Natural Sciences, Biomedical Engineering Department, Bahçeşehir University, Istanbul 34353, Turkey

**Keywords:** intracranial hemorrhage, computed tomography, medical image segmentation, Attention U-Net, deep supervision, test-time augmentation, artificial intelligence, RSNA, BHSD

## Abstract

**Background/Objectives:** Intracranial hemorrhage is a life-threatening condition that requires rapid and accurate diagnosis using computed tomography (CT). Manual segmentation is time-consuming and subject to interobserver variability, motivating the development of automated segmentation methods. This study proposes an enhanced Attention U-Net for intracranial hemorrhage segmentation using the RSNA Intracranial Hemorrhage Detection dataset, in which only 1082 of 20,154 images have pixel-level annotations. The proposed architecture integrates attention gates, deep supervision, cosine annealing, and test-time augmentation. An ablation study was also conducted to evaluate the contribution of each component. **Methods:** The proposed Attention U-Net and a baseline U-Net were trained and evaluated using identical experimental protocols. Performance was assessed on an independent test set using the Dice score, IoU, sensitivity, and precision. To evaluate the reproducibility of the performance advantage across datasets, both architectures were additionally trained and evaluated under identical protocols on the independent Brain Hemorrhage Segmentation Dataset (BHSD), comprising 2961 annotated axial slices derived from 192 CT volumes. The BHSD test set consisted of 621 slices. **Results:** On the independent RSNA test set, the proposed Attention U-Net achieved a Dice score of 0.7965 (±0.0814), outperforming the baseline U-Net (0.7683 ± 0.0906). The proposed model also achieved an IoU of 0.6689, sensitivity of 0.8094, and precision of 0.7966. On the BHSD test set, the Attention U-Net again outperformed the baseline U-Net, achieving a Dice score of 0.5676 versus 0.5063, sensitivity of 0.5350 versus 0.5138, and IoU of 0.4408 versus 0.3924. Thus, the proposed architecture demonstrated a consistent relative performance advantage across both independent datasets, although absolute performance was lower on BHSD than on RSNA. **Conclusions:** The findings demonstrate that incorporating attention mechanisms, multiscale supervision, and inference-time enhancement can improve intracranial hemorrhage segmentation performance. The consistent improvement over the baseline U-Net across two independent datasets further supports the effectiveness and cross-dataset robustness of the proposed architecture. The results also highlight the potential of semi-supervised learning to leverage the large volume of unlabeled CT data available in intracranial hemorrhage datasets.

## 1. Introduction

Intracranial hemorrhage (ICH) refers to the extravascular accumulation of blood within the cranial cavity and is a serious neurological condition with potentially life-threatening consequences. ICH has diverse etiologies, including traumatic brain injury, hypertensive hemorrhage, vascular malformations, intracranial tumors, cerebral venous thrombosis, and cerebral amyloid angiopathy [1]. Because of its heterogeneous presentation and potential for rapid progression, the timely and accurate detection of ICH is critical for effective clinical management and improved patient outcomes.

The estimated incidence of ICH is approximately 25 cases per 100,000 person-years, with a mortality rate reaching nearly 40% within the first month after onset [2]. Timely and accurate neuroimaging is therefore essential for determining the location and volume of the hemorrhage, assessing the risk of secondary cerebral injury, and guiding urgent treatment decisions.

Computed tomography (CT) is the gold-standard imaging modality for the evaluation of acute brain injuries due to its rapid acquisition time, wide availability, and high sensitivity to hemorrhagic lesions [3]. CT imaging is essential for identifying time-sensitive abnormalities such as intracranial hemorrhage, midline shift, and skull fractures. Among hemorrhage subtypes, intraparenchymal hemorrhage (IPH), subdural hematoma (SDH), and epidural hematoma (EDH) may produce significant mass effect and often require immediate neurosurgical intervention. Therefore, accurate detection and segmentation of hemorrhagic regions in CT scans are critical for assessing hemorrhage volume, guiding treatment strategies, and improving patient outcomes.

In current clinical practice, radiologists manually identify hemorrhagic lesions on CT images. This process is time-consuming and subject to interobserver variability and human error. To address these limitations, computer-aided diagnosis (CAD) systems have been increasingly investigated, with artificial intelligence (AI) playing a central role in automating medical-image analysis [4]. Automatic segmentation is a fundamental component of CAD pipelines because it provides precise localization and delineation of hemorrhagic regions for diagnosis and clinical interpretation.

Most existing studies have focused on binary discrimination between hemorrhagic and non-hemorrhagic regions [5], while fewer works have addressed accurate pixel-level segmentation of hemorrhagic areas. Moreover, reported segmentation performance in the literature remains limited, particularly in cases with irregular boundaries or complex anatomical structures. Therefore, there is a continued need for robust and precise segmentation methods.

Among deep learning architectures, the U-Net [6] convolutional neural network has become one of the most widely adopted models for biomedical image segmentation. U-Net is specifically designed for pixel-wise prediction and combines contextual information with fine spatial details through encoder–decoder structures and skip connections. Although previous studies have demonstrated the potential of deep learning for ICH analysis, many have focused on hemorrhage subtype classification or volumetric quantification rather than precise pixel-level segmentation.

Motivated by the clinical need for accurate ICH delineation in low-contrast CT images, we propose a unified attention-guided segmentation framework. The model addresses severe foreground–background imbalance and boundary ambiguity associated with small, irregular hemorrhagic lesions. Multiscale attention mechanisms are integrated into an encoder–decoder architecture to enhance lesion-relevant representations while suppressing irrelevant background activations.

To improve optimization stability during training, we use a cosine-annealing learning-rate schedule that promotes smoother convergence and reduces oscillations in the gradient updates. We also introduce auxiliary supervision at intermediate decoder stages to strengthen gradient propagation and support multiscale feature learning.

During inference, prediction consistency is enhanced through test-time augmentation, in which predictions obtained from geometrically transformed versions of each input are inverse-transformed and averaged. This procedure improves robustness and reduces prediction variance.

A controlled ablation study is conducted to evaluate the contribution of each component. All models are assessed using Dice coefficient, precision, sensitivity, specificity, IoU, and F1-score. To further probe whether these gains reproduce beyond a single dataset, both architectures are additionally retrained and re-evaluated, without modification, on the independent BHSD dataset.

To the best of our knowledge, this study is among the first to combine attention mechanisms, deep supervision, cosine annealing, and test-time augmentation (TTA) within a unified framework for intracranial hemorrhage segmentation. The resulting design is compact and provides improved segmentation performance.

## 2. Related Work

### 2.1. ICH Detection and Classification

Considerable research has been conducted on the automated diagnosis of intracranial hemorrhage (ICH). Most existing studies focus either on binary detection, in which the presence of ICH is identified, or on multiclass classification, in which different ICH subtypes are recognized.

Early approaches relied on traditional machine learning techniques using handcrafted features. For instance, Yuh et al. [7] proposed a threshold-based algorithm that detects ICH subtypes based on location, shape, and volume, achieving high sensitivity (98%) but relatively low specificity (59%). Similarly, Li et al. [8] developed methods combining atlas-based registration and Bayesian classifiers, achieving high sensitivity and accuracy on small datasets.

With the advancement of deep learning, convolutional neural networks (CNNs) became the dominant approach. Prevedello et al. [9] proposed CNN-based models for detecting ICH and related abnormalities, achieving an AUC of 0.91. Chilamkurthy et al. [10] utilized a large-scale dataset and a ResNet-based architecture combined with a random forest classifier, reporting an average AUC of 0.93 for ICH subtype classification.

To incorporate inter-slice dependencies, hybrid CNN-RNN architectures have been introduced. Grewal et al. [11] combined DenseNet with bidirectional LSTM, achieving improved detection performance and an F1-score of 0.84. Similarly, Ye et al. [12] proposed a 3D CNN-RNN framework to detect and classify ICH regions.

### 2.2. ICH Segmentation

Accurate segmentation of ICH is crucial for clinical decision-making, particularly for estimating hemorrhage volume. Traditional segmentation methods relied on preprocessing steps such as skull stripping, noise removal, and feature extraction, followed by clustering or contour-based techniques. For example, Prakash et al. [13] and Shahangian et al. [14] employed level-set methods (DRLSE) to delineate hemorrhage regions, achieving Dice scores up to 0.88. Other approaches utilized fuzzy c-means clustering and wavelet-based thresholding, reporting Dice scores around 0.82 [15].

Recent work has shifted toward deep-learning-based segmentation. CNN-based and fully convolutional network (FCN) architectures enable pixel-wise prediction and have substantially improved performance. Chang et al. [16] proposed a region-based CNN for joint detection and segmentation and achieved an average Dice score of 0.85. Kuo et al. [17] introduced a patch-based FCN framework with active learning and reported high precision for both detection and segmentation.

Other studies employed architectures such as U-Net and its variants, which have become the standard in medical image segmentation. Wang et al. [18] developed a human–computer interaction approach to improve diagnostic accuracy, while Yu et al. [19] applied U-Net with dimensionality reduction for volume measurement. Nijiati et al. [20] proposed a symmetric Transformer-based network achieving a Dice score of 0.716 ± 0.031. Peng et al. [21] introduced AttFocusNet with attention mechanisms, achieving high accuracy (Dice up to 0.91).

#### 2.2.1. RSNA-Based Segmentation Studies

Several recent studies have utilized the RSNA 2019 Brain CT Hemorrhage Dataset due to its large scale and diversity. However, since the dataset originally provides only slice-level labels, many researchers have extended it with manual or semi-automatic annotations to enable segmentation tasks.

A notable study by Yüce et al. [22] proposed a comprehensive framework for the automatic segmentation and volumetric analysis of ICH using 3D deep-learning models. Their dataset comprised 1508 CT volumes from the RSNA dataset, QURE500, and a hospital dataset. Expert radiologists manually annotated multiple structures, including brain parenchyma, cerebrospinal fluid, edema, and several hemorrhage subtypes. The authors trained multiple 3D U-Net models and evaluated them using five-fold cross-validation. Their best model achieved a Dice score of approximately 0.70 for ICH segmentation and a specificity of 82%. However, its performance was affected by the complexity of multiclass segmentation and the presence of small hemorrhages. Ramananda and Sundaresan [23] proposed a weakly supervised framework based on the RSNA dataset and achieved Dice scores of 0.583 and 0.640 in low-data settings.

In another related work [24], voxel-level annotated datasets derived from RSNA were used to evaluate multiple 3D architectures, including U-Net, V-Net, Attention U-Net, and nnU-Net. Among these, nnU-Net achieved the best performance with a Dice score close to 0.79, demonstrating the effectiveness of automated configuration strategies.

#### 2.2.2. BHSD-Based Segmentation Studies

The Brain Hemorrhage Segmentation Dataset (BHSD) [25] is a more recently introduced 3D multi-class benchmark comprising 192 CT volumes with voxel-level annotations spanning five hemorrhage subtypes (EDH, IPH, IVH, SAH, and SDH), together with a further 1980 volumes of unannotated reconstructed data. Because it provides genuine voxel-level ground truth rather than derived polygon annotations, BHSD has recently been adopted as a challenging benchmark for both fully and semi-supervised ICH segmentation. Zhu et al. [26] evaluated several supervised and semi-supervised baselines on BHSD using 96 labeled and 500 unlabeled volumes: a fully supervised nnU-Net achieved a Dice score of 45.10 ± 0.21, while semi-supervised strategies such as Mean-Teacher, Interpolation Consistency, and Cross Pseudo Supervision improved this to 44.63, 45.38, and 49.50, respectively. Their proposed SAM-2-based semi-supervised method, SSS, achieved a state-of-the-art Dice score of 53.15 ± 0.20 on the same benchmark. The reported Dice scores on BHSD, ranging from approximately 0.45 to 0.53, reflect the challenges associated with intracranial hemorrhage segmentation on an independent dataset. The limited number of fully annotated samples available in BHSD may make robust pixel-level representation learning more challenging. Furthermore, differences in annotation protocols and dataset characteristics may influence the absolute segmentation performance.

### 2.3. Discussion of Related Work

Despite significant progress, several limitations remain in existing works. Many studies rely on small datasets, limiting generalization capability. While 3D deep learning models capture volumetric context, they introduce high computational cost and require extensive annotations. Additionally, multi-class classification and segmentation tasks remain challenging, particularly for small hemorrhage regions.

### 2.4. Position of This Work

In contrast to prior methods, this study introduces a unified attention-guided framework for accurately segmenting small hemorrhagic lesions. We developed an enhanced 2D U-Net that integrates attention mechanisms, deep supervision, and test-time augmentation. The proposed model was evaluated against a standard four-level U-Net under identical experimental conditions, and an ablation study was used to quantify the incremental contribution of each component. Beyond the primary RSNA-based evaluation, we further assess the transferability of the proposed enhancements by repeating the identical baseline-versus-proposed comparison on the independent BHSD dataset, providing, to our knowledge, one of the first controlled cross-dataset comparisons of attention-gated 2D U-Nets for ICH segmentation.

## 3. Materials and Methods

### 3.1. Dataset and Expert Annotation

This study utilizes the RSNA Intracranial Hemorrhage Detection dataset [27], a large-scale publicly available collection of brain CT scans comprising 20,154 images acquired across multiple clinical sites. The dataset was originally released as a classification benchmark providing only slice-level labels indicating the presence or absence of five hemorrhage subtypes (epidural, intraparenchymal, intraventricular, subarachnoid, and subdural). Critically, no pixel-level segmentation annotations are provided for any image in the original release, rendering the dataset unsuitable for supervised segmentation training without additional expert annotation effort.

#### 3.1.1. Expert Pixel-Level Annotation

A fundamental contribution of this work is the construction of a pixel-level segmentation dataset derived from the RSNA collection. Because the original RSNA release provides only slice-level classification labels and no pixel-level segmentation ground truth, the annotated subset used in this study was constructed purposively rather than through random sampling. Radiologists reviewed RSNA CT images and manually delineated hemorrhagic regions in images in which intracranial hemorrhage was visually identified. Of the images submitted for annotation, 1421 received usable polygon annotations. During quality control, 339 images were excluded because the hemorrhage mask covered more than 40% of the image area; manual review confirmed these cases were annotation/rasterization artifacts (e.g., masks spanning the entire slice) rather than valid lesion delineations, so they were removed to avoid introducing unreliable ground truth. This quality-control filter yielded the final set of 1082 valid image–mask pairs used in this study. Expert radiologists manually annotated 1082 CT images with precise polygon-based delineations of hemorrhagic regions. Annotation was performed at the binary level: for each image, radiologists delineated all hemorrhagic pixels as a single foreground class, without separately labeling individual hemorrhage subtypes (EDH, IPH, IVH, SAH, and SDH) at the pixel level. This binary annotation protocol was adopted because subtype-resolved pixel-level delineation substantially increases annotation time per image, and because the large majority of prior ICH segmentation studies likewise formulate the task as binary segmentation. As a direct consequence, the dataset does not support a statistically reliable per-subtype breakdown of Dice or sensitivity, nor a report of the proportion of samples belonging to each hemorrhage subtype; both are identified as priority directions for future work (Section 5.2). The annotation process was conducted using the Roboflow platform (version 1.0) [28], which provided a structured environment for polygon labeling, multi-stage quality control, and annotation management. All annotations were reviewed and validated by the participating radiologists to ensure clinical accuracy. The finalized annotations were exported in COCO JSON format and used to construct the segmentation dataset for model training and evaluation.

Pixel-level delineation of hemorrhagic regions is clinically demanding because it requires specialized neuroradiological expertise, substantial time for each image, and iterative quality control. This annotation burden helps explain why the RSNA dataset, despite containing more than 20,000 images, has rarely been used for pixel-level segmentation. Previous RSNA-based segmentation studies have either used low-data or weakly supervised settings [23] or supplemented the dataset with annotations from additional clinical sources [22]. To the best of our knowledge, the 1082 annotated image–mask pairs created in this study constitute one of the largest pixel-level ICH segmentation datasets derived exclusively from the RSNA collection. This dataset contribution may support future segmentation research beyond the scope of the present study.

For this study, all hemorrhage subtypes were treated as a single foreground class, formulating the task as binary segmentation of hemorrhagic versus non-hemorrhagic tissue, consistent with the binary annotation protocol described above. This design choice reflects the clinical priority of rapid hemorrhage detection and delineation, irrespective of subtype, and is consistent with several prior segmentation works [16,19]. The remaining 19,072 images in the dataset carry only slice-level classification labels and were not used for supervised segmentation training; leveraging this large unannotated pool through semi- or self-supervised learning is identified as a priority direction for future work.

**Figure 1:**
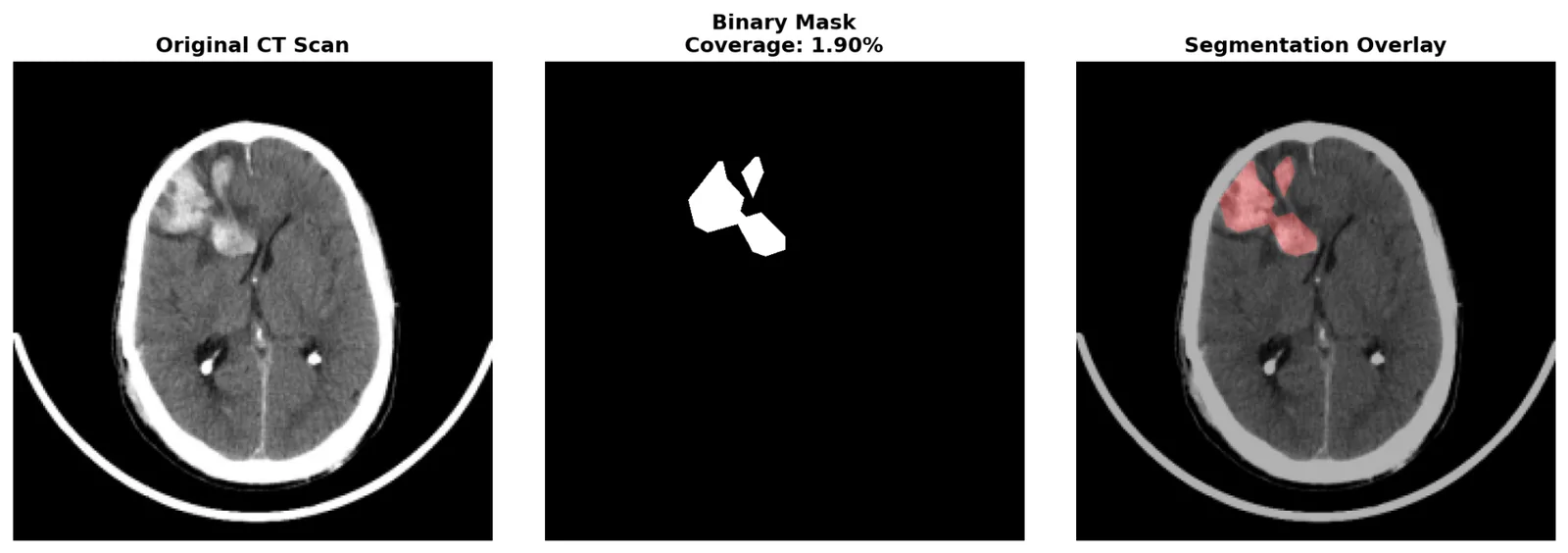
Representative ICH segmentation case from the RSNA dataset. (**Left**) original brain CT slice. (**Center**) binary segmentation mask with a hemorrhage coverage of 1.90%. (**Right**) segmentation overlay. The mask was produced by expert radiologists using polygon-based annotation on the Roboflow platform, with quality control applied at each stage.

Figure 1 displays a representative annotated case from the dataset, illustrating the original CT slice, the binary hemorrhage mask, and the segmentation overlay.

#### 3.1.2. Data Splitting Strategy

The 1082 valid pairs were partitioned into three mutually exclusive subsets using random sampling with a fixed random seed (42) to ensure reproducibility across experimental runs:

- Training set: 756 samples (69.9%)—used for model parameter optimization.
- Validation set: 109 samples (10.1%)—used exclusively for hyperparameter tuning and checkpoint selection.
- Test set: 217 samples (20.1%)—held out entirely until final evaluation; not used in any model selection decision.

The split was performed in two stages: the test set was first separated (20%), and the remaining data were then divided into training and validation subsets. No image appearing in the test set was used during training or validation, ensuring image-level separation between model development and performance assessment.

### 3.2. Secondary Evaluation Dataset: BHSD

To assess whether the performance gains obtained on RSNA reproduce on an independently collected and independently annotated cohort, we additionally trained and evaluated both architectures on the Brain Hemorrhage Segmentation Dataset (BHSD) Unlike RSNA, which required post hoc polygon annotation on the Roboflow platform (Section 3.1.1), BHSD provides genuine expert-generated voxel-level 3D segmentation masks for 192 CT volumes, covering the same five hemorrhage subtypes as RSNA (EDH, IPH, IVH, SAH, and SDH). A further 1980 unannotated volumes of reconstructed data were not used in this fully supervised experiment.

#### 3.2.1. 3D-to-2D Slice Extraction

Each of the 192 annotated BHSD 3D CT volumes was decomposed into individual axial 2D slices to match the 2D segmentation architecture used in this study. All hemorrhage subtypes were merged into a single binary foreground class, consistent with the binary segmentation protocol adopted for the RSNA dataset. To reduce background imbalance, empty slices without hemorrhagic pixels were down-sampled, while near-empty slices containing negligible brain tissue were excluded. This preprocessing yielded 2961 usable annotated 2D slices for training and evaluation.

#### 3.2.2. Patient-Level Data Splitting

To prevent information leakage between slices from the same CT volume, the BHSD dataset was partitioned at the patient level using a 70/10/20 split. The training, validation, and test sets comprised 133, 20, and 39 patients, respectively, corresponding to 2035, 305, and 621 2D slices. No slices from patients in the test set were included in the training or validation sets.

#### 3.2.3. Preprocessing and Training Protocol

Identical preprocessing was applied to BHSD and RSNA: grayscale conversion, intensity normalization to [0, 1], and channel-dimension expansion to (1,H,W), with no data augmentation. Both the baseline U-Net (Section 3.4.1) and the proposed Attention U-Net with deep supervision, cosine annealing, and TTA (Section 3.4.2) were retrained from randomly initialized weights on the BHSD training set, using the identical hyperparameters, loss function (Equations (5) and (8)), optimizer, batch size (8), and epoch budget (25) reported in Table 1 for the RSNA experiments, with AMP enabled throughout. No architectural or hyperparameter tuning specific to BHSD was performed; this design choice was deliberate, so that any observed change in relative model ranking or absolute performance can be attributed to properties of the dataset itself rather than to additional tuning effort. Although BHSD is originally provided as a 3D volumetric CT dataset, the present validation experiment uses the same 2D segmentation framework as the RSNA experiments. Each annotated 3D CT volume was therefore decomposed into individual axial 2D slices before training and evaluation. Consequently, the model does not exploit inter-slice 3D contextual information, which represents a limitation of the adopted 2D architecture and is discussed further in Section 5.2.

### 3.3. Preprocessing and Data Preparation

#### 3.3.1. Mask Generation from COCO Annotations

Pixel-level segmentation masks were generated deterministically from the COCO-format polygon annotations. For each CT image, polygon vertices were converted to integer pixel coordinates and rasterized into binary masks using the fillPoly function from the OpenCV library. Hemorrhagic regions were assigned a pixel value of 255, and the background was set to 0, producing 8-bit grayscale binary masks saved in lossless PNG format for storage efficiency and pipeline compatibility. The rasterization procedure is fully deterministic given the polygon coordinates, ensuring that mask generation can be exactly reproduced from the original COCO JSON annotations.

#### 3.3.2. Image Normalization

CT images were preprocessed prior to network input through the following standardized pipeline:

1. Grayscale conversion: images were loaded as single-channel 8-bit grayscale arrays, discarding redundant color channels present in some DICOM-derived PNG exports.
2. Intensity normalization: pixel intensities were scaled to the continuous range [0, 1] by dividing by 255, ensuring numerical stability during gradient-based optimization and compatibility with sigmoid output activations.
3. Channel dimension expansion: a singleton channel dimension was added to produce tensors of shape (1,H,W), consistent with PyTorch’s version 2.11.0+cu130 Conv2d input convention.

No data augmentation was applied during training. This conservative choice was made to avoid introducing label inconsistencies into the polygon-derived masks through inappropriate geometric transformations. The effect of carefully selected augmentation strategies on segmentation performance will be investigated in future work.

### 3.4. Network Architectures

We design and evaluate two encoder–decoder convolutional architectures for pixel-wise binary segmentation of intracranial hemorrhage in CT images. Both architectures share an identical backbone depth and channel progression (1→64→128→256→512→1024), ensuring that observed performance differences are attributable solely to the proposed architectural and training innovations rather than to differences in model capacity or depth.

#### 3.4.1. Baseline: Standard U-Net (Four Encoder Levels)

The baseline model is a faithful implementation of the U-Net architecture of Ronneberger et al. [6], retaining the full four-level encoder–decoder structure and 1024-channel bottleneck of the original formulation.

##### Encoder

The encoding path consists of four successive downsampling stages. Each stage applies a *DoubleConv* block—two sequential 3×3 convolutions each followed by Batch Normalization and a Rectified Linear Unit (ReLU) activation—which extracts increasingly abstract feature representations. Spatial resolution is halved after each stage by a 2×2 MaxPooling operation with stride 2. The channel dimension doubles at every level, 1→64→128→256→512, yielding hierarchical feature maps that encode both fine-grained local texture and coarser semantic context.

##### Bottleneck

A final DoubleConv block at the deepest resolution maps the encoder output from 512 to 1024 channels, providing the richest abstract representation before decoding begins.

##### Decoder

The decoding path symmetrically inverts the encoder through four successive upsampling stages (1024→512→256→128→64). At each stage, a 2×2 transposed convolution doubles the spatial resolution and halves the channel count. The upsampled feature map is concatenated with the spatially corresponding encoder feature map via a skip connection, which re-introduces fine spatial detail suppressed during downsampling, and a DoubleConv block refines the concatenated representation.

##### Output Head

A 1×1 convolution projected the 64-channel decoder output onto a single-channel map. A sigmoid activation is then applied to produce pixel-wise hemorrhage probabilities in the interval [0, 1]. Batch normalization is applied at every convolutional stage to stabilize training and accelerate convergence.

#### 3.4.2. Proposed Model: Attention U-Net with Deep Supervision and Test-Time Augmentation

The proposed model extends the standard four-level U-Net baseline by incorporating four complementary and independently motivated enhancements: (i) soft additive attention gates applied to all skip connections, (ii) deep supervision through auxiliary decoder heads, (iii) test-time augmentation (TTA) during inference, and (iv) a cosine annealing learning rate schedule. The encoder–decoder backbone remains identical to the baseline, ensuring a fully controlled setting for fair comparison. This design allows the individual contribution of each component to be systematically evaluated. Each enhancement addresses a specific limitation of the conventional U-Net, as discussed below.

(i) Soft Additive Attention Gates.

Standard U-Net skip connections concatenate all encoder activations with the corresponding decoder map, irrespective of their spatial relevance to the target class. In hemorrhage segmentation, where the foreground typically occupies a small (often < 5%) and spatially irregular fraction of each CT slice, this indiscriminate aggregation introduces substantial irrelevant background signal into the decoder, impeding precise localization of hemorrhagic boundaries.

To address this limitation, each of the four skip connections was equipped with a Soft Additive Attention Gate (AG) based on the formulation of Oktay et al. [29]. Each AG is a lightweight self-gating module that learns to amplify hemorrhage-relevant activations and suppress background responses without requiring an additional supervision signal or a region-proposal mechanism. This mechanism enables the network to emphasize relevant spatial regions without explicitly cropping a region of interest between layers.

Formally, let $x_{i}^{l}\in R^{F_{l}}$ denote the encoder skip feature vector at pixel *i* in layer *l*, and let $g_{i}\in R^{F_{g}}$ denote the gating signal from the coarser decoder level, which carries richer semantic context but coarser spatial detail. The attention gate computes a scalar attention coefficient $\alpha_{i}^{l}\in \left[0,1\right]$ for each spatial position via an additive compatibility function:

(1) $$q_{\mathrm{att}}^{l}=\psi^{⊤}\;\left(\sigma_{1}\;\left(W_{x}^{⊤}\;x_{i}^{l}+W_{g}^{⊤}\;g_{i}+b_{g}\right)\right)+b_{\psi }$$

(2) $$\alpha_{i}^{l}=\sigma_{2}\;\left(q_{\mathrm{att}}^{l}\;\left(x_{i}^{l};\;g_{i};\;\Theta_{\mathrm{att}}\right)\right)$$

where $W_{x}\in R^{F_{l}\times F_{\mathrm{int}}}$, $W_{g}\in R^{F_{g}\times F_{\mathrm{int}}}$, and $\psi \in R^{F_{\mathrm{int}}\times 1}$ are learned 1×1 convolution kernels that project inputs to a shared intermediate space of dimension $F_{\mathrm{int}}$; $b_{g}\in R^{F_{\mathrm{int}}}$ and $b_{\psi }\in R$ are bias terms; $\sigma_{1}=\mathrm{ReLU}$ introduces non-linearity; and $\sigma_{2}=\sigma \left(\cdot \right)$ is the sigmoid activation that constrains $\alpha_{i}^{l}$ to the unit interval. The set of learnable parameters is $\Theta_{\mathrm{att}}=\left\{W_{x},W_{g},\psi ,b_{g},b_{\psi }\right\}$.

The attended skip features passed to the decoder are obtained by element-wise (Hadamard) multiplication of the attention map with the encoder feature map:

(3) $${\hat{x}}_{i}^{l}=\alpha_{i}^{l}\cdot x_{i}^{l}$$

broadcast across all $F_{l}$ channels at position *i*. The gate converges to assign $\alpha_{i}^{l}\approx 1$ over hemorrhagic foreground regions and $\alpha_{i}^{l}\approx 0$ over background, effectively implementing a soft spatial mask trained jointly with the segmentation objective without any external supervision.

In our implementation, the gating signal $g_{i}$ may be spatially coarser than the skip features $x_{i}^{l}$; it is therefore bilinearly upsampled to match the skip resolution before computing Equation (1). The intermediate dimension is set to $F_{\mathrm{int}}=F_{l}/2$ at every level, yielding $F_{\mathrm{int}}\in \left\{32,64,128,256\right\}$ for decoder levels 1 through 4, respectively. All linear transformations are realized as channel-wise 1×1 convolutions without spatial support, keeping the number of additional trainable parameters minimal.

The update rule for convolution parameters in layer l−1 receiving attended features from layer *l* is as follows [29]:

(4) $$\frac{𝜕\;\left(\alpha_{i}^{l}\;f\;\left(x_{i}^{l-1};\Phi^{l-1}\right)\right)}{𝜕\;\left(\Phi^{l-1}\right)}=\alpha_{i}^{l}\frac{𝜕f\;\left(x_{i}^{l-1};\Phi^{l-1}\right)}{𝜕\;\left(\Phi^{l-1}\right)}+\frac{𝜕\;\left(\alpha_{i}^{l}\right)}{𝜕\;\left(\Phi^{l-1}\right)}x_{i}^{l-1}$$

The first gradient term is scaled by $\alpha_{i}^{l}$, so that gradients from background regions are down-weighted during backpropagation. This allows parameters in shallower encoder layers to be updated primarily based on spatial regions that are clinically relevant to hemorrhage localization.

(ii) Deep Supervision.

Supervision propagated solely through the final output layer must traverse all decoder stages before reaching early encoder–decoder interface layers, making gradients susceptible to vanishing in deeper architectures. Deep supervision [30,31] addresses this by attaching auxiliary output heads at intermediate decoder levels, providing direct short-circuit gradient paths to earlier parameters and ensuring that intermediate feature maps remain semantically discriminative at each image scale [29].

Auxiliary heads are attached to the feature maps produced after the DoubleConv blocks at decoder levels $u_{4}$, $u_{3}$, and $u_{2}$. Each head consists of a 1×1 convolution mapping intermediate features to a single-channel prediction map, followed by bilinear upsampling to the input image resolution and a sigmoid activation. The total training loss is a weighted linear combination of the main Dice loss and the three auxiliary Dice losses:

(5) $$L_{\mathrm{total}}=L_{\mathrm{main}}+\lambda_{4}\;L_{u_{4}}+\lambda_{3}\;L_{u_{3}}+\lambda_{2}\;L_{u_{2}}$$

with weights $\lambda_{4}=0.4$, $\lambda_{3}=0.3$, and $\lambda_{2}=0.2$, which decay with decoder depth to reflect the increasing spatial coarseness of shallower feature maps. This weighting scheme prioritizes the main output prediction while ensuring non-trivial gradient signals propagate to all supervised decoder levels. At inference, all auxiliary heads are discarded; only the main output is used for prediction.

(iii) Test-Time Augmentation.

A single forward pass may exhibit sensitivity to left–right or top–bottom spatial orientation, particularly for hemorrhages located near image borders or presenting irregular morphology. Test-time augmentation mitigates this by aggregating predictions from geometrically transformed copies of each test input, producing a consensus estimate that is more robust to spatial biases introduced by the training data distribution.

At inference, each test image x undergoes three forward passes through the trained network $f_{\theta }$: the original orientation, a horizontal flip $F_{h}$, and a vertical flip $F_{v}$. Each flipped prediction is inverse-transformed before averaging:

(6) $$\hat{p}=\frac{1}{3}\left(f_{\theta }\left(x\right)\;+\;F_{h}\;\left[f_{\theta }\;\left(F_{h}\left(x\right)\right)\right]\;+\;F_{v}\;\left[f_{\theta }\;\left(F_{v}\left(x\right)\right)\right]\right)$$

Horizontal and vertical flips are realized via torch.flip along the width (dim = 3) and height (dim = 2) dimensions, respectively, with the inverse flip applied to each prediction before averaging to restore spatial correspondence with the original input. Since TTA operates exclusively at inference and requires no additional training, it constitutes a zero-cost performance improvement applicable to any trained segmentation model.

(iv) Cosine Annealing Learning Rate Schedule.

A fixed learning rate risks premature stagnation when set too high—gradients oscillate around sharp loss minima, as evidenced by the Dice collapse to 0.2885 observed at epoch 17 in the baseline U-Net under a fixed learning rate—or slow convergence when set too low. Cosine annealing [32] provides a principled decay schedule that smoothly reduces the learning rate following a half-cosine curve from an initial value $\eta_{\max}$ to a minimum $\eta_{\min}$ over $T_{\max}$ epochs:

(7) $$\eta_{t}=\eta_{\min}+\frac{1}{2}\left(\eta_{\max}-\eta_{\min}\right)\left(1+\cos\;\left(\frac{t\;\pi }{T_{\max}}\right)\right)$$

where *t* is the current epoch index. We set $\eta_{\max}=10^{-4}$, $\eta_{\min}=10^{-6}$, and $T_{\max}=25$. This schedule enables the Attention U-Net to reach its best validation checkpoint three epochs earlier than the baseline (epoch 22 vs. epoch 25), while avoiding the oscillatory behavior observed under fixed learning rate.

### 3.5. Loss Function

Both models are trained using a soft Dice loss function, which directly optimizes the spatial overlap between predicted and ground-truth regions and is well suited for handling the severe foreground–background class imbalance inherent in CT hemorrhage segmentation, where hemorrhagic pixels typically constitute less than 5% of each image. For a predicted probability map p and binary ground-truth mask g, the loss for a single image is as follows:

(8) $$L_{\mathrm{Dice}}\left(p,\;g\right)=1-\frac{2\sum_{i}p_{i}\;g_{i}+\varepsilon }{\sum_{i}p_{i}+\sum_{i}g_{i}+\varepsilon }$$

where $p_{i}\in \left[0,1\right]$ is the predicted sigmoid probability at pixel *i*, $g_{i}\in \left\{0,1\right\}$ is the binary ground-truth label, and ε=1.0 is a Laplace smoothing term that prevents numerical instability when both the prediction and the ground truth are entirely empty (i.e., no hemorrhage is present). Unlike cross-entropy loss, the Dice loss directly penalizes the overlap deficit between predicted and ground-truth regions and is intrinsically robust to class imbalance without requiring explicit class weighting. For the Attention U-Net, $L_{\mathrm{Dice}}$ is applied independently to the main output and to each auxiliary head as defined in Equation (5).

### 3.6. Optimization and Training Configuration

Both models are optimized using the Adam optimizer with an initial learning rate $\eta =10^{-4}$, momentum parameters $\beta_{1}=0.9$, $\beta_{2}=0.999$, and numerical stability constant $\epsilon =10^{-8}$. All experiments use a batch size 8 with 4 parallel DataLoader workers, with pin_memory and persistent_workers enabled to minimize CPU-to-GPU data transfer latency. Automatic mixed-precision (AMP) training was employed to improve computational efficiency.

Training was performed for 25 epochs on a single NVIDIA CUDA-enabled GPU. For final test evaluation, we retained the checkpoint with the highest validation Dice score. Model selection was therefore based exclusively on the validation set, without access to the test labels.

The Attention U-Net additionally employs the cosine annealing schedule described in Equation (7), with $T_{\max}=25$ and $\eta_{\min}=10^{-6}$. The baseline U-Net is trained with a fixed learning rate throughout. All other hyperparameters, data splits, loss functions, and hardware conditions are held constant between the two models to ensure a fully controlled and reproducible comparison. The identical protocol, unchanged, was subsequently applied to the BHSD dataset (Section 3.2).

Table 1 summarizes the complete configuration for both architectures.

### 3.7. Evaluation Metrics

Model performance was assessed using seven complementary metrics computed from the binary confusion matrix elements (TP, TN, FP, and FN) at a fixed decision threshold of 0.5. This threshold was chosen to maintain comparability with prior ICH segmentation literature and was not tuned on the test set. The evaluation metrics used in this study are as follows:

The Dice Similarity Coefficient (DSC) measures the harmonic mean of precision and recall at the pixel level and serves as the primary evaluation metric. It is defined as follows:

(9) $$\mathrm{DSC}=\frac{2\;\mathrm{TP}}{2\;\mathrm{TP}+\mathrm{FP}+\mathrm{FN}}$$

Pixel Accuracy is defined as follows:

(10) $$\mathrm{Accuracy}=\frac{\mathrm{TP}+\mathrm{TN}}{\mathrm{TP}+\mathrm{TN}+\mathrm{FP}+\mathrm{FN}}.$$

Precision measures the proportion of predicted hemorrhage pixels that are true hemorrhage:

(11) $$\mathrm{Precision}=\frac{\mathrm{TP}}{\mathrm{TP}+\mathrm{FP}}$$

Sensitivity (Recall) is the clinically most critical metric in this context, as it measures the proportion of true hemorrhage pixels correctly identified. Missed hemorrhages (false negatives) carry direct clinical risk.

(12) $$\mathrm{Sensitivity}=\frac{\mathrm{TP}}{\mathrm{TP}+\mathrm{FN}}$$

Specificity is defined as follows:

(13) $$\mathrm{Specificity}=\frac{\mathrm{TN}}{\mathrm{TN}+\mathrm{FP}}$$

F1 Score is computed directly from the same confusion-matrix counts as DSC:

(14) $$F1=\frac{2\;\mathrm{TP}}{2\;\mathrm{TP}+\mathrm{FP}+\mathrm{FN}}$$

Under this definition, F1 is numerically identical to DSC (Equation (9)) for both models on both datasets.

Intersection over Union (IoU/ Jaccard Index), provides a stricter measure of spatial overlap than DSC by penalizing false positives and false negatives equally:

(15) $$\mathrm{IoU}=\frac{\mathrm{TP}}{\mathrm{TP}+\mathrm{FP}+\mathrm{FN}}$$

### 3.8. Implementation Details

Both models were implemented in PyTorch 2.x (Python 3.x). Image loading and mask rasterization used OpenCV 4.x and NumPy 2.1.3. Training and inference were performed on a single NVIDIA CUDA-enabled GPU with automatic mixed-precision enabled throughout. Segmentation predictions on validation and test sets were visualized using a four-color scheme: yellow for true positives, red for false positives, green for false negatives, and gray for true negatives, enabling rapid qualitative assessment of prediction errors. Validation prediction visualizations were saved every five epochs to monitor training progression. All experiments are fully reproducible from the fixed random seed, deterministic data splits, and the implementation described above.

## 4. Results

### 4.1. Dataset Composition

Table 2 presents the final dataset distribution after the annotation and data preparation procedures. The final segmentation dataset comprised 1082 CT images with corresponding expert-annotated polygon masks, which were used for model training, validation, and testing.

### 4.2. Baseline U-Net Performance

The standard four-level U-Net was trained for 25 epochs with a batch size 8 using 95 training batches per epoch, with AMP enabled. The best validation Dice score of 0.7696 was achieved at epoch 25. Table 3 summarizes the final test set performance.

### 4.3. Ablation Study

To isolate the contribution of each architectural and training innovation, we conducted a systematic ablation study by training three intermediate model variants, each adding one component incrementally over the baseline. All variants share the same encoder–decoder backbone, dataset split (random seed 42), batch size, and AMP configuration. The results reported below are from the independent test set of 217 samples.

#### 4.3.1. Variant A: U-Net + Cosine Annealing LR Only

The first ablation variant retains the plain U-Net architecture (no attention gates, no deep supervision, and no TTA) but introduces the cosine annealing learning rate schedule (Equation (7)). The best validation Dice of 0.7818 was achieved at epoch 25. Test set results are shown in Table 4.

Compared to the fixed-LR baseline, adding cosine annealing alone yields a Dice improvement of +0.0079 (0.7683 → 0.7762) and a sensitivity gain of +0.0296 (0.7582 → 0.7878), confirming that smoother learning rate decay stabilizes optimization and reduces oscillatory behavior.

#### 4.3.2. Variant B: U-Net + Cosine LR + Attention Gates

The second variant adds soft additive attention gates on all four skip connections while retaining the cosine annealing schedule. Deep supervision and TTA are not yet applied. The best validation Dice of 0.7868 was achieved at epoch 25. Test set results are shown in Table 5.

Adding attention gates to Variant A leads to a further Dice gain of +0.0048 (0.7762 → 0.7810). Notably, the standard deviation of Dice scores decreases from ±0.0910 to ±0.0803, indicating that attention gates reduce inter-sample prediction variability by suppressing irrelevant background activations. Precision also improves from 0.7791 to 0.7858 (+0.0067), reflecting fewer false positive detections.

#### 4.3.3. Variant C: U-Net + Cosine LR + Attention + Deep Supervision (No TTA)

The third variant further adds deep supervision (auxiliary heads at decoder levels $u_{4}$, $u_{3}$, and $u_{2}$ with loss weights 1.0/0.4/0.3/0.2), while TTA is still withheld. The best validation Dice of 0.7802 was achieved at epoch 25. Test set results are shown in Table 6.

Adding deep supervision produces the largest single-component gain in sensitivity: from 0.7877 (Variant B) to 0.8128 (+0.0251), reaching a sensitivity above 0.80 on the RSNA test set even without TTA. The Dice coefficient improves by +0.0121 (0.7810 → 0.7931), and IoU improves by +0.0162 (0.6474 → 0.6636). The auxiliary short-circuit gradient paths provided by deep supervision are particularly effective for suppressing false negatives, as the multi-scale training signals force intermediate decoder layers to maintain spatial awareness of hemorrhagic regions at all resolutions.

### 4.4. Attention U-Net Performance (Full Proposed Model)

The full proposed Attention U-Net, which combined cosine annealing, attention gates, deep supervision, and TTA, was trained for 25 epochs with AMP enabled, a batch size of 8, and 95 training batches per epoch. The highest validation Dice score, 0.7833, was achieved at epoch 22. During test evaluation, TTA was applied using the original image, a horizontal flip, and a vertical flip. The final test results are summarized in Table 7.

### 4.5. Model Comparison

Table 8 provides a direct comparison between the two primary architectures across all seven evaluation metrics.

Table 9 consolidates the ablation study results, showing the incremental contribution of each component to the final model’s performance.

**Figure 2:**
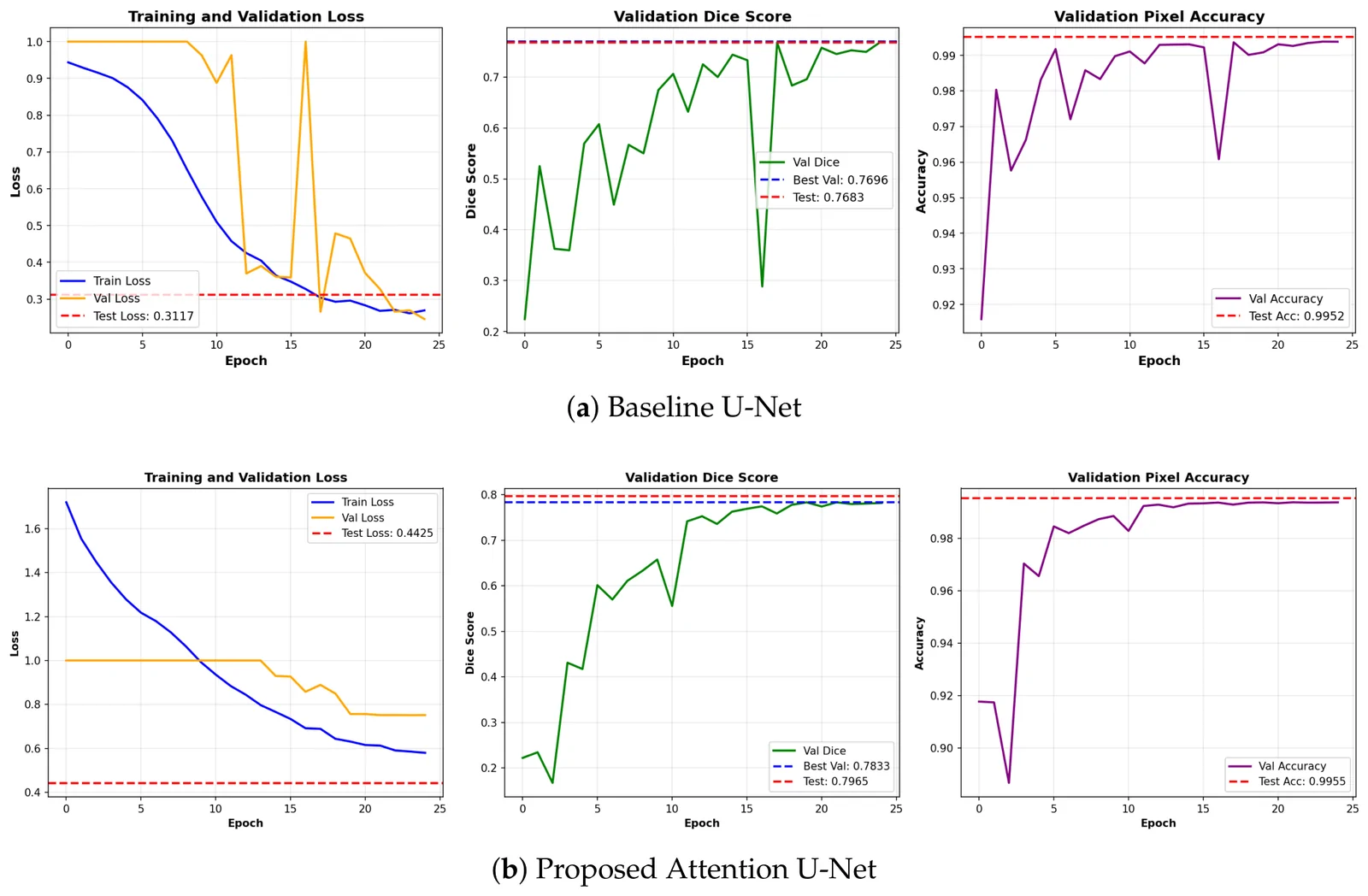
Training and validation loss, Dice score, and accuracy curves over 25 epochs for both models. The proposed Attention U-Net demonstrates faster convergence, reduced oscillations, and improved generalization compared to the baseline U-Net.

**Figure 3:**
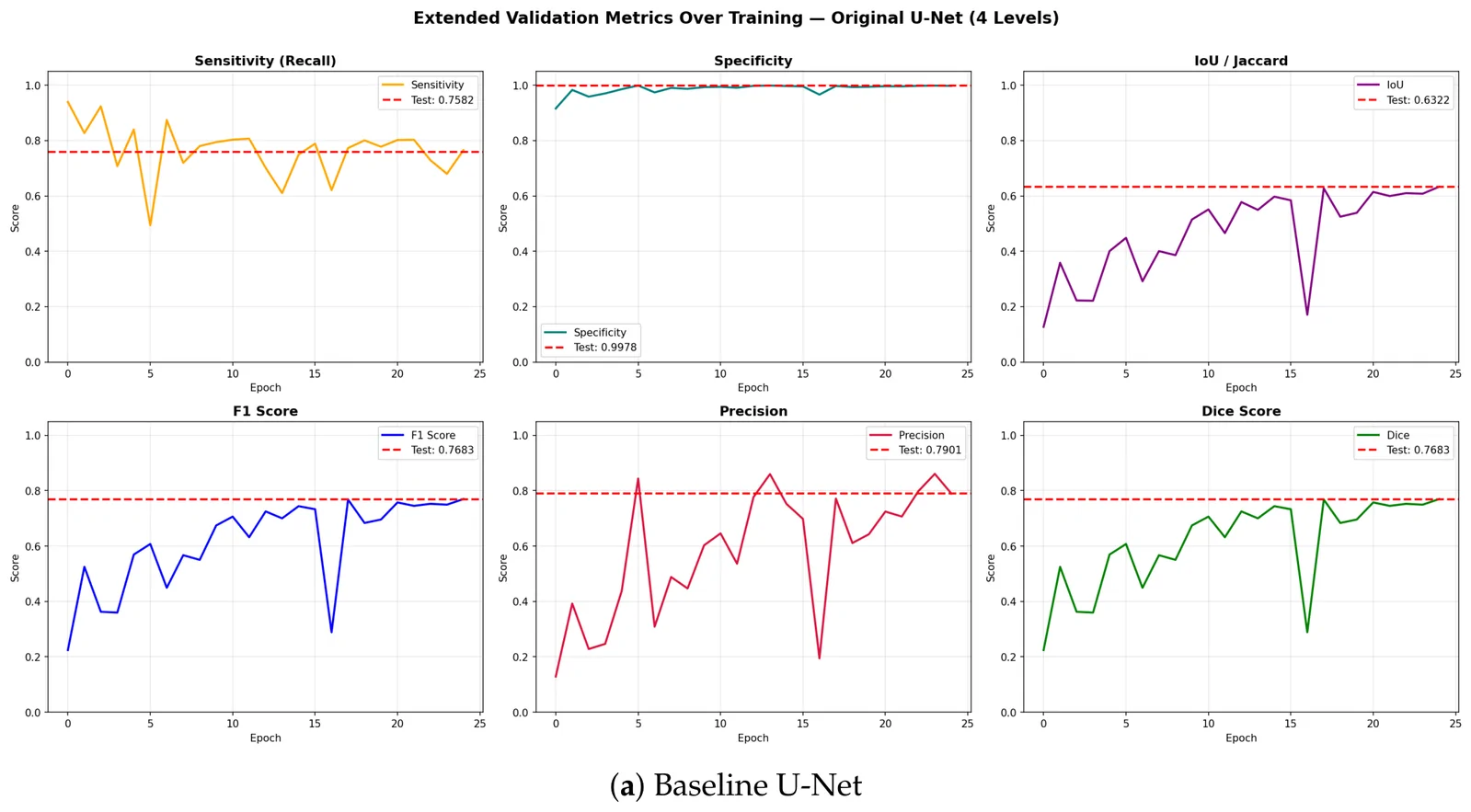

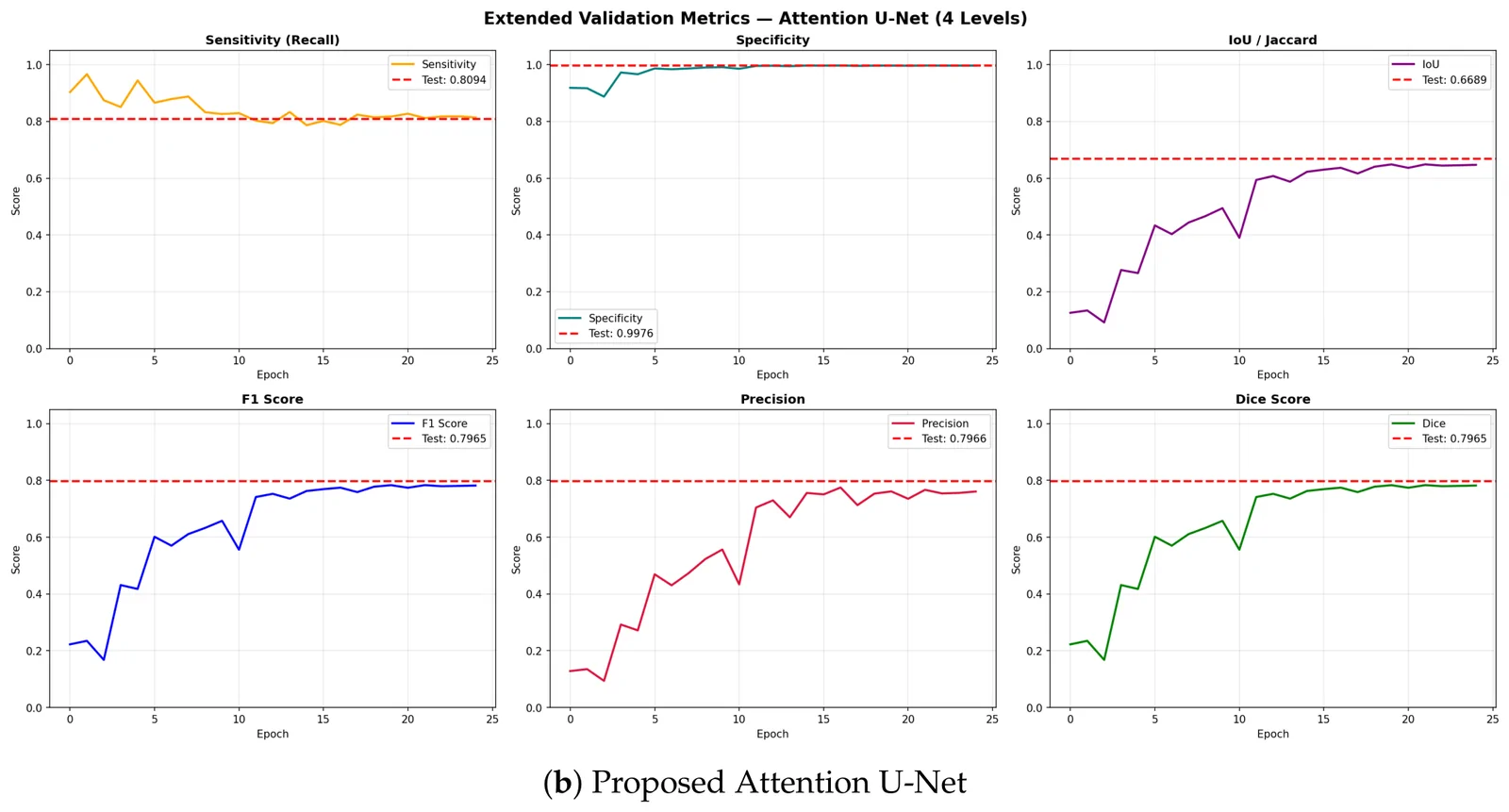
Per-epoch evolution of precision, sensitivity, specificity, IoU, F1-score, and Dice coefficient for both models during training. The Attention U-Net consistently shows higher sensitivity and lower variability compared to the baseline U-Net across all epochs.

Figure 2 shows the training and validation loss and Dice score curves for both models. Figure 3 presents the per-epoch evolution of additional metrics.

### 4.6. Overall Performance Summary

**Figure 4:**
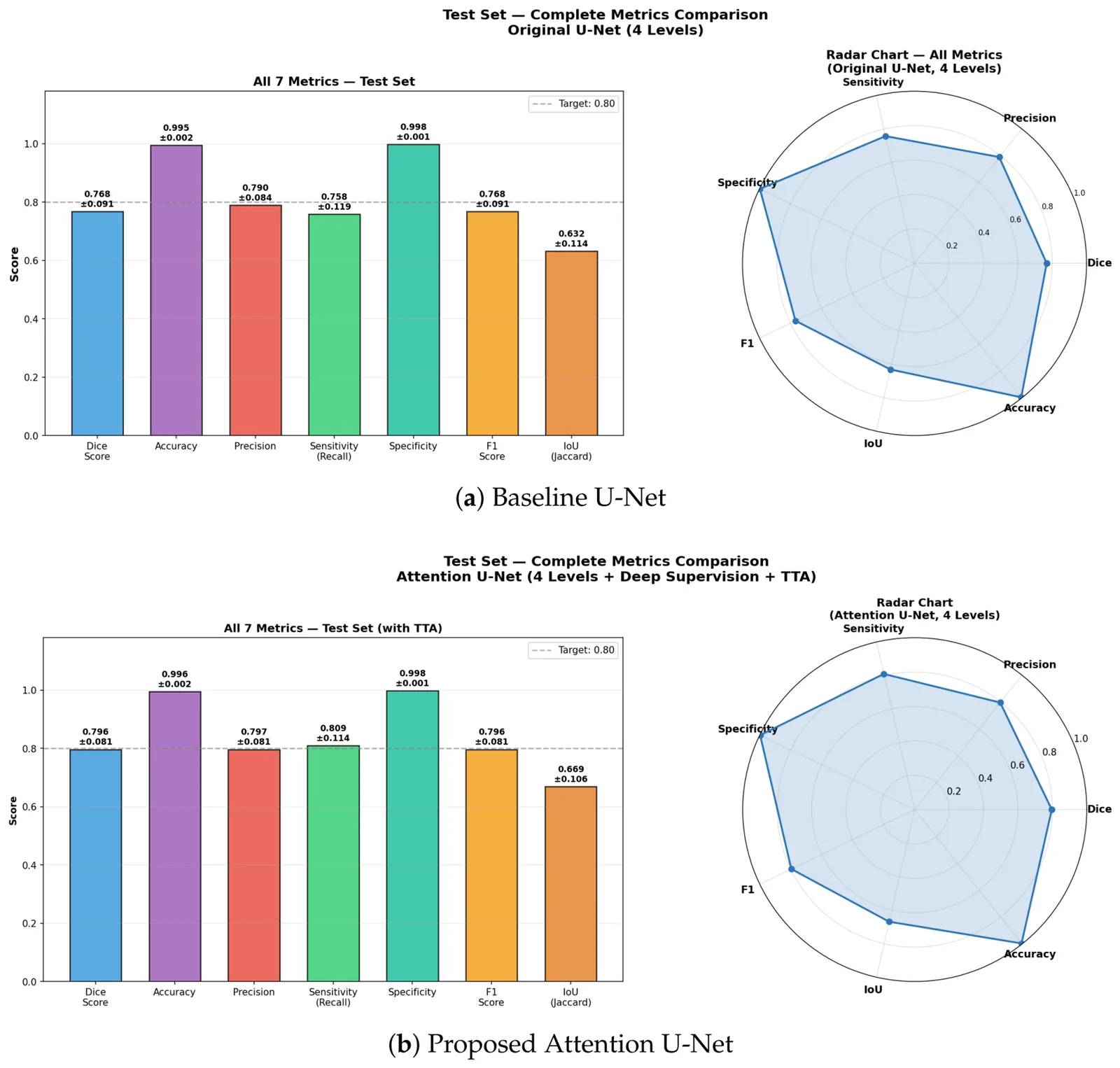
Comparison of test-set segmentation performance across seven evaluation metrics: Dice, IoU, precision, sensitivity, specificity, F1 score, and pixel accuracy. The dashed line marks the 0.80 reference value used for benchmarking only. The proposed Attention U-Net outperformed the baseline on six of the seven metrics, while the baseline achieved marginally higher specificity.

Figure 4 compares the baseline U-Net and the proposed Attention U-Net across the seven evaluation metrics on the test set. The proposed model achieved higher Dice, IoU, precision, sensitivity, F1 score, and pixel accuracy, whereas specificity was marginally lower.

### 4.7. Performance Analysis

#### 4.7.1. Segmentation Accuracy

The Attention U-Net achieved a Dice coefficient of 0.7965, a gain of +0.0282 over the baseline U-Net (0.7683). The standard deviation of Dice scores across test batches decreased from ±0.0906 (U-Net) to ±0.0814 (Attention U-Net), indicating that attention mechanisms in combination with deep supervision and TTA produce more consistent predictions across heterogeneous hemorrhage presentations.

#### 4.7.2. Sensitivity and Clinical Relevance

The most clinically significant improvement is in sensitivity: the Attention U-Net achieves 0.8094 vs. 0.7582 for the baseline (Δ = +0.0512). Sensitivity measures the proportion of actual hemorrhage pixels correctly identified; an increase of 5.12 percentage points directly translates to a reduction in missed hemorrhage detections. Critically, the Attention U-Net is the only one of the two primary models (baseline and proposed) to exceed the illustrative 0.80 sensitivity reference used here for model comparison; this value is not a clinically validated diagnostic cutoff and should not be interpreted as such without prospective clinical validation.

The ablation study indicated that the sensitivity gain was driven primarily by deep supervision. Variant C, which did not use TTA, achieved a sensitivity of 0.8128 and therefore exceeded the 0.80 reference value described above. Adding TTA reduced sensitivity slightly to 0.8094 but further increased the Dice score and IoU. These findings suggest that deep supervision mainly reduced false-negative predictions, whereas TTA primarily improved overall spatial overlap and prediction robustness.

#### 4.7.3. Precision, Specificity, and IoU

Precision improved marginally from 0.7901 to 0.7966 (+0.0065), indicating slightly fewer false positive detections per positive prediction. Specificity was ≥0.9974 for both models, confirming reliable rejection of non-hemorrhagic background with negligible difference between architectures. The IoU improved from 0.6322 to 0.6689 (+0.0367), reflecting tighter spatial agreement between predicted and ground truth segmentation contours.

#### 4.7.4. Training Convergence

The Attention U-Net achieved its best validation Dice score (0.7833) at epoch 22, whereas the U-Net continued improving until epoch 25, before plateauing at 0.7696. The faster convergence is attributed to both the stronger gradient signal provided by deep supervision and the smooth learning rate decay of cosine annealing, which avoids the abrupt oscillations observed in the U-Net training curve (notably the Dice drop to 0.2885 at epoch 17 under fixed learning rate). Table 10 reports the generalization gap between validation and test performance for both models.

**Figure 5:**
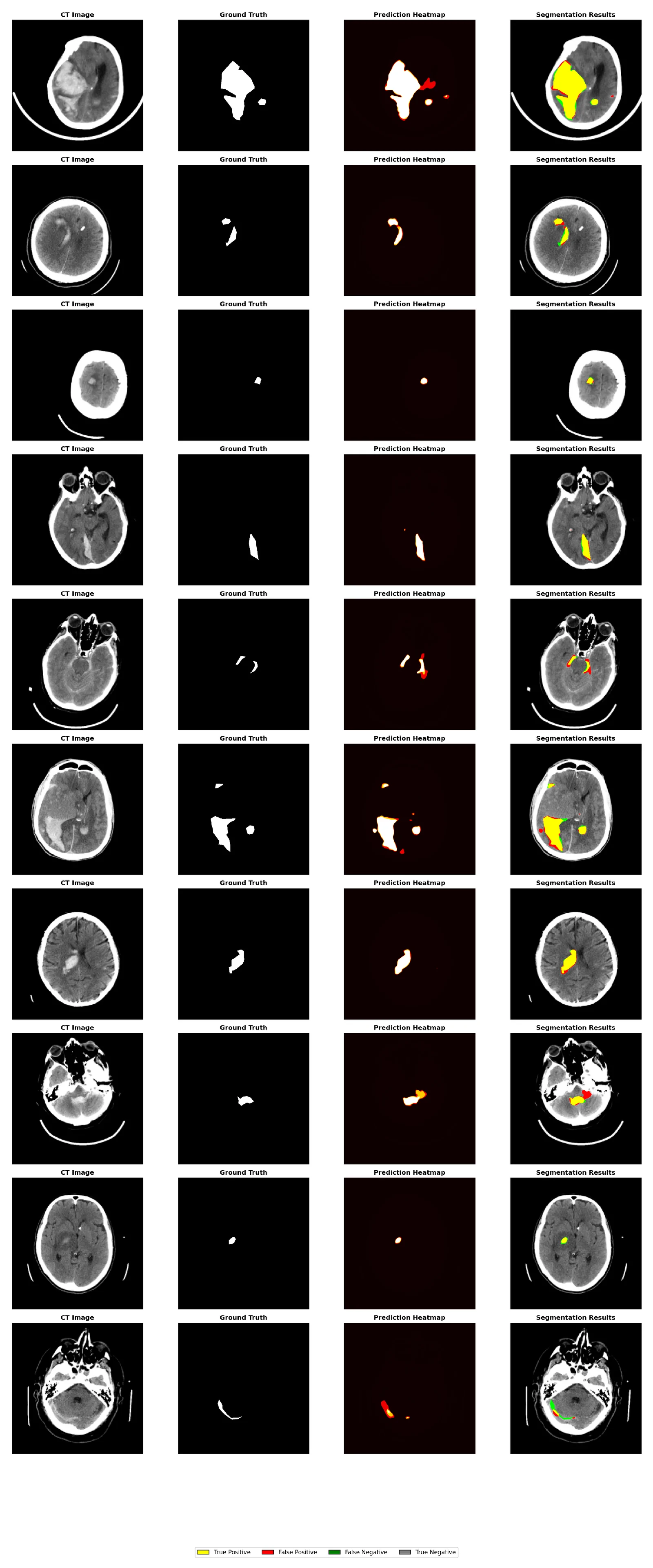
Color-coded segmentation predictions from the Attention U-Net on test set CT images. Yellow: true positives; red: false positives; green: false negatives; gray: true negatives. Ground truth masks are shown for reference.

The Attention U-Net achieved a test Dice score of 0.7965, which was 0.0132 higher than its best validation Dice score of 0.7833. If the generalization gap is defined as Test Dice minus Validation Dice, the gap is therefore +0.0132 for the Attention U-Net and −0.0013 for the baseline U-Net. The higher test score for the proposed model may partly reflect the use of TTA during test evaluation, because TTA was not applied when the validation checkpoint was selected; however, differences between the validation and test subsets may also have contributed. Representative color-coded test predictions of the Attention U-Net are shown in Figure 5.

### 4.8. Cross-Dataset Reproducibility Assessment on BHSD

To determine whether the performance advantage of the proposed Attention U-Net is reproducible beyond RSNA, both architectures were retrained from scratch and evaluated on the independent BHSD cohort, described in Section 3.2, under the identical protocol reported in Table 1.

#### 4.8.1. BHSD Dataset Composition

Table 11 summarizes the resulting slice-level dataset after 3D-to-2D conversion and patient-level splitting.

#### 4.8.2. Baseline U-Net Performance on BHSD

The baseline U-Net, trained under a fixed learning rate as in the RSNA experiments, reached its best validation Dice score of 0.5226, at epoch 14. Table 12 reports the corresponding test set performance (621 samples).

#### 4.8.3. Attention U-Net Performance on BHSD

The proposed Attention U-Net (attention gates + deep supervision + cosine annealing + TTA) achieved a best validation Dice score of 0.5931 at epoch 18, higher than the baseline’s 0.5226. At test time, TTA was applied using the original image, a horizontal flip, and a vertical flip. Test set results (621 samples) are reported in Table 13.

#### 4.8.4. BHSD Model Comparison

Table 14 directly compares the baseline U-Net and the proposed Attention U-Net on BHSD across all seven evaluation metrics, mirroring Table 8 for RSNA.

**Figure 6:**
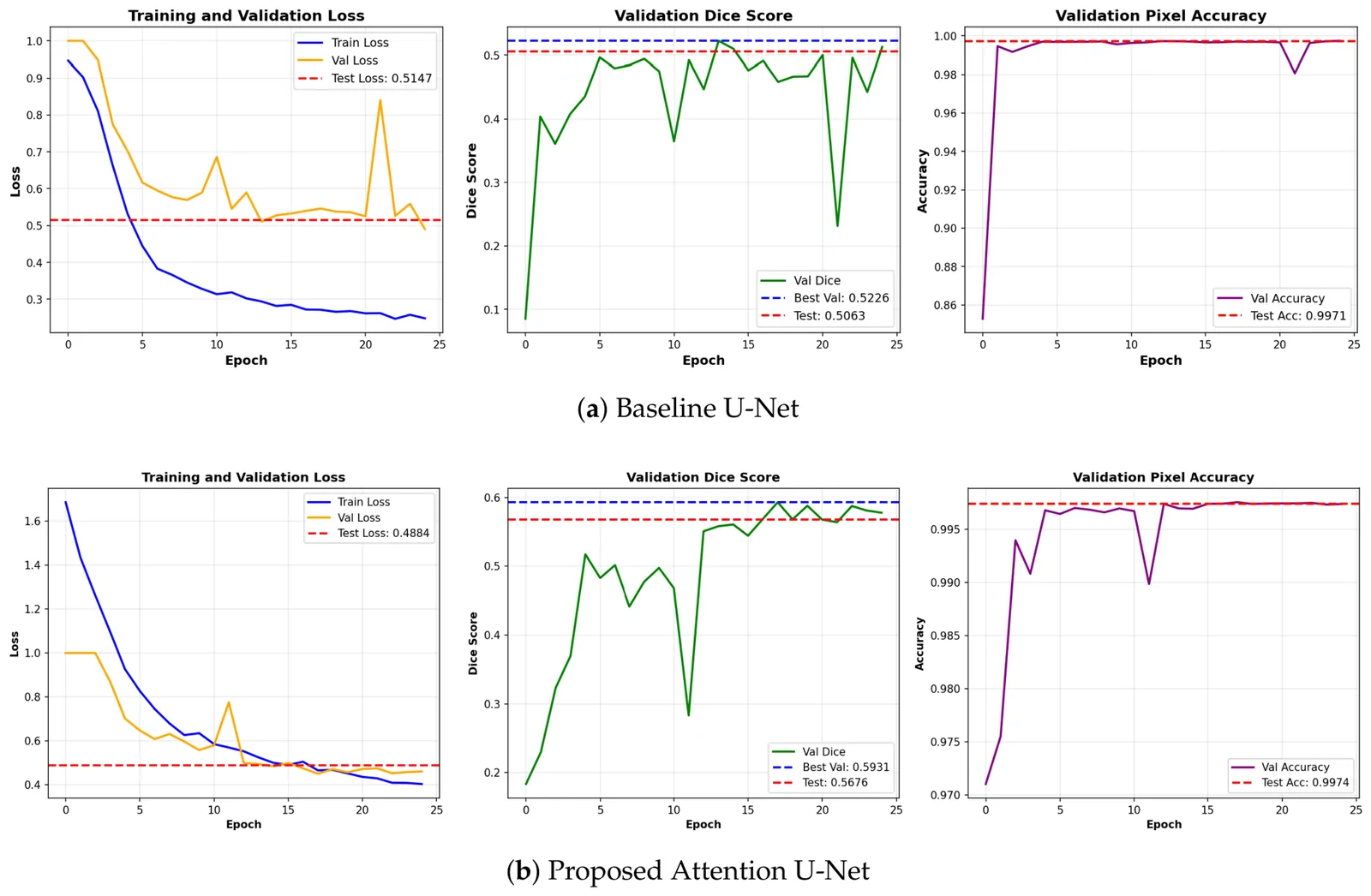
BHSD training and validation loss, together with validation Dice score and pixel accuracy, are presented over 25 epochs for both models. The validation curves exhibit noticeable epoch-to-epoch variability, particularly for Dice and validation loss, while pixel accuracy remains consistently high.

**Figure 7:**
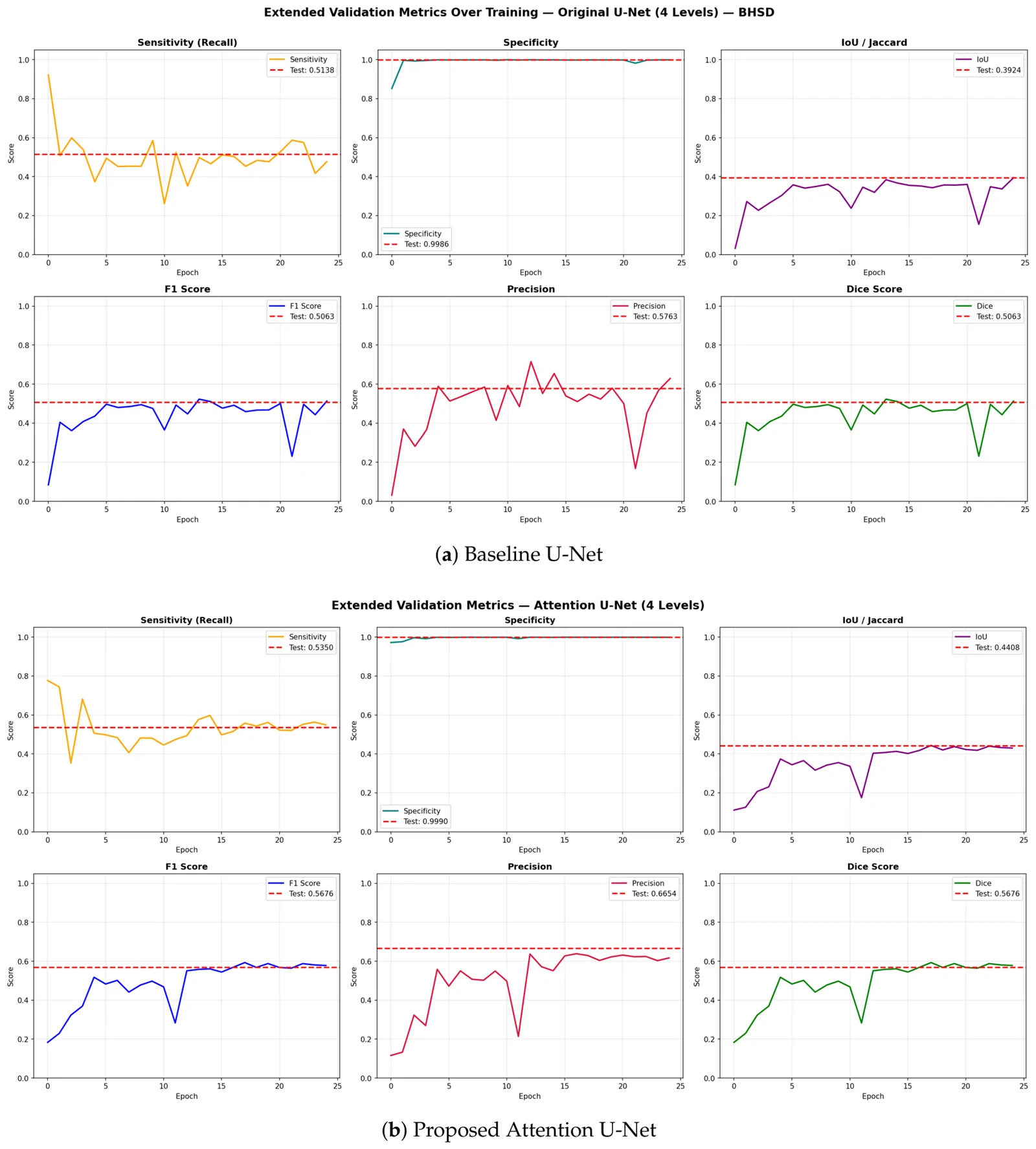
Per-epoch evolution of sensitivity, specificity, IoU, F1-score, precision, and Dice coefficient on BHSD validation data for both models, with final test-set values overlaid as dashed reference lines.

**Figure 8:**
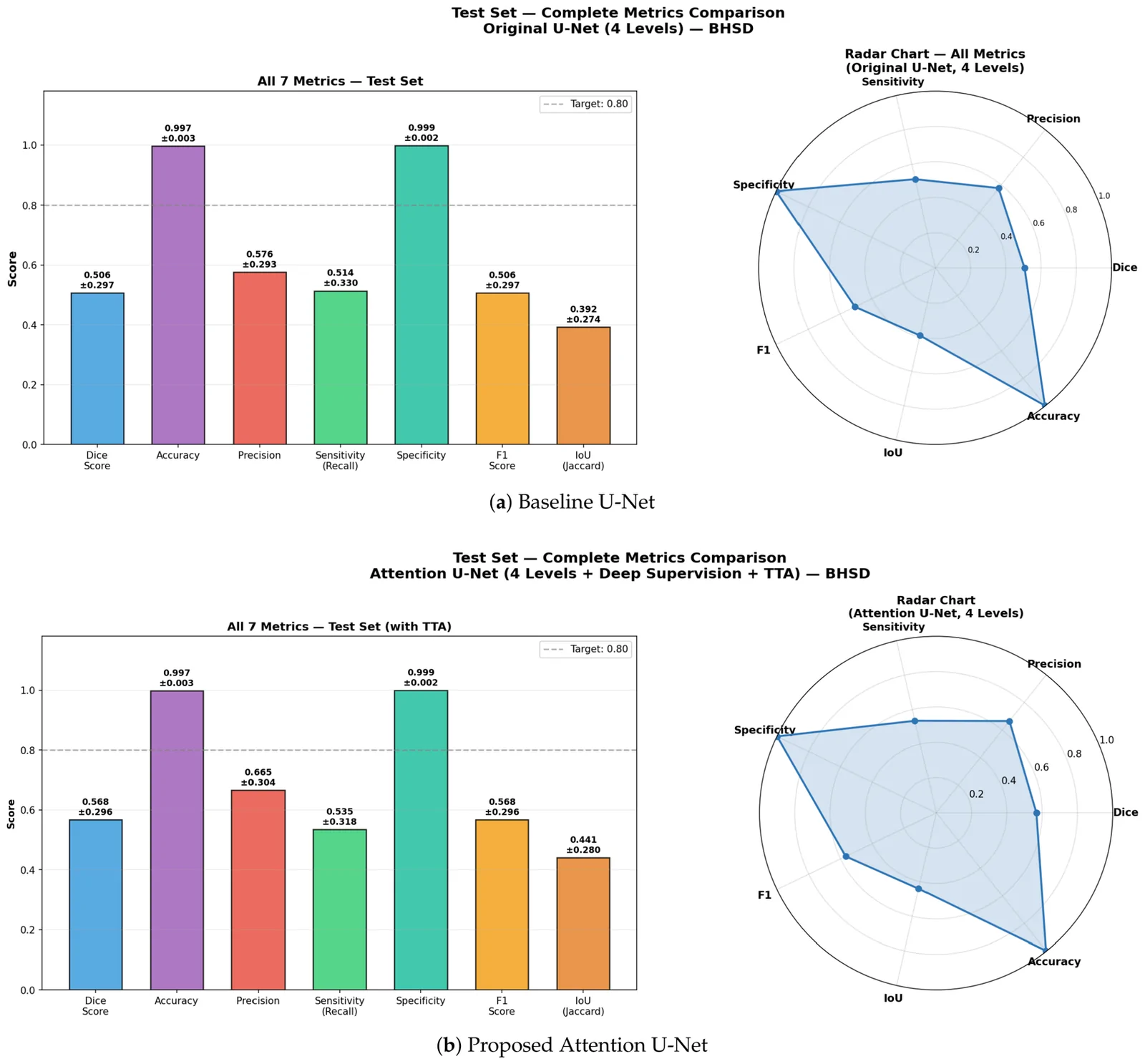
BHSD test-set performance across all seven evaluation metrics for both models, shown as bar charts and radar plots. The proposed Attention U-Net outperforms the baseline on Dice, accuracy, precision, sensitivity, specificity, F1 score, and IoU.

On every point-estimate metric, the Attention U-Net outperformed the baseline U-Net on BHSD, reproducing the consistent performance advantage observed on RSNA (Table 8). The improvements in Dice, sensitivity, and IoU on BHSD (+0.0613, +0.0212, and +0.0484, respectively) further support the effectiveness of the proposed architecture on an additional independent dataset. The BHSD training and validation curves are shown in Figure 6, the per-epoch evolution of the evaluation metrics in Figure 7, and the final test-set metrics in Figure 8.

#### 4.8.5. Paired Statistical Comparison

To provide a rigorous, non-inflated statistical comparison, we conducted paired analyses on both datasets, using per-case Dice rather than the batch-level Dice reported in Table 8 and Table 14. Specifically, Dice was computed separately for each test image (RSNA, n=217) or each test patient (BHSD, n=39, obtained by averaging across that patient’s slices); this per-case unit is the appropriate one for paired statistical testing, since both models are evaluated on the same cases, whereas the batch-level figures in the comparison tables are not directly comparable to it because Dice is a non-linear ratio. For RSNA, per-image Dice was compared for the 217 test images (pairing is valid here regardless of the image-level split, since each image is scored by both models); for BHSD, per-patient mean Dice was compared across the 39 test patients, exploiting the dataset’s true patient-level split. On RSNA, the Attention U-Net achieved a mean paired improvement of 0.0193 over the baseline (95% bootstrap CI, 10,000 resamples: [0.0056, 0.0336]; two-sided Wilcoxon signed-rank test, p=0.0335). On BHSD, the mean paired improvement was 0.0514 (95% bootstrap CI, 10,000 resamples: [0.0221, 0.0842]; two-sided Wilcoxon signed-rank test, p=0.000168). Both results are statistically significant at α=0.05, confirming that the Attention U-Net’s advantage over the baseline is not attributable to chance on either dataset.

#### 4.8.6. Comparison with External BHSD Literature

Table 15 situates our BHSD results relative to previously reported methods evaluated on the same underlying 192-volume annotated BHSD cohort. Because these studies differ substantially in supervision regime (fully vs. semi-supervised), the amount of labeled data used, and network dimensionality (2D vs. 3D), the comparison is provided for context only and should not be interpreted as a strictly controlled benchmark.

Although the proposed models use a comparatively simple 2D architecture and do not rely on unlabeled data, the full proposed Attention U-Net (56.76%) exceeded the 53.15% reported for the semi-supervised SSS method of [26] on BHSD, whereas the baseline U-Net (50.63%) did not reach this benchmark. This comparison should, however, be interpreted with caution because the experimental settings differ substantially. Our models were trained using the 133 annotated volumes in the training split, corresponding to 2035 axial 2D slices after 3D-to-2D conversion, while the remaining 20 and 39 volumes were reserved for validation and independent testing, respectively. In contrast, [26] used a label-scarce semi-supervised setting with 96 labeled volumes and 500 unlabeled volumes. Therefore, the two approaches address different research questions: our study evaluates fully supervised segmentation using the available annotated training data, whereas [26] investigates the benefit of unlabeled data under limited annotation. The results nevertheless demonstrate that the proposed framework achieves competitive performance on the independent BHSD test set while consistently outperforming the baseline U-Net under the same experimental protocol.

## 5. Discussion

The proposed Attention U-Net, enhanced with attention gates, deep supervision, test-time augmentation, and cosine annealing, demonstrates improved performance over the standard U-Net for ICH segmentation. In particular, it achieves higher sensitivity (0.7582 → 0.8094) and an improved Dice score (0.7683 → 0.7965), indicating more accurate detection and segmentation of hemorrhagic regions.

The ablation study provides quantitative evidence for the incremental contribution of each component. Cosine annealing alone yields a Dice improvement of +0.0079 and reduces training instability. Attention gates contribute an additional +0.0048 Dice gain and notably reduce prediction variability (Dice std: ±0.0910 →±0.0803), consistent with their role in suppressing irrelevant background activations in the skip connections. Deep supervision produces the most substantial individual gain, with sensitivity increasing by +0.0251 (from 0.7877 to 0.8128 without TTA), attributable to the auxiliary gradient paths strengthening the detection of small or boundary-adjacent hemorrhagic regions. Finally, TTA at inference raises Dice by a further +0.0034 (0.7931 → 0.7965) and IoU by +0.0053 (0.6636 → 0.6689) with no additional training cost, confirming its effectiveness as a zero-overhead ensemble strategy.

The limited availability of annotated data (5.4%) is attributed to the high cost of expert pixel-level labeling, as the RSNA dataset was originally designed for classification rather than segmentation tasks.

A comparison with previous RSNA-based studies provides additional context. Yüce et al. [22] used a 3D deep-learning framework trained on RSNA-derived volumetric data combined with additional datasets and expert annotations and reported a Dice score of approximately 0.70 for ICH segmentation. Ramananda and Sundaresan [23] reported Dice scores of 0.583–0.640 in low-data, weakly supervised settings using the same dataset. Song et al. [24] reported that nnU-Net achieved a Dice score of approximately 0.79 on voxel-annotated RSNA data with full 3D volumetric context. The proposed 2D Attention U-Net achieved a Dice score of 0.7965, which is competitive with this reported 3D result (Table 16).

### 5.1. Cross-Dataset Reproducibility: RSNA vs. BHSD

The BHSD experiments provide an independent assessment of cross-dataset reproducibility. The relative ranking of the models was preserved, with Attention U-Net consistently outperforming the baseline U-Net across all point-estimate metrics, as observed on RSNA. This agreement is particularly informative because the RSNA split was performed at the image level and cannot fully exclude patient overlap between subsets (Section 5.2), whereas the BHSD split was strictly patient-level; the BHSD result therefore provides leakage-free support for the direction of the effect, even though the RSNA point estimates themselves should be interpreted with that caveat in mind. However, absolute performance was lower on BHSD, with Dice scores of 0.5063–0.5676 compared with 0.7683–0.7965 on RSNA. This difference may reflect the more detailed voxel-level 3D annotations in BHSD, the smaller number of unique patients, and the removal of empty slices during 2D dataset construction, which may reduce anatomical diversity and contextual information. Finally, the proposed 2D Attention U-Net achieved 56.76% Dice, exceeding the 53.15% reported for the semi-supervised 3D SAM-2-based method of [26]. Since the two studies differ in supervision strategy and labeled-data availability, this comparison should be interpreted as contextual rather than a direct head-to-head evaluation.

### 5.2. Limitations

This study has four main limitations. First, the RSNA-derived annotated subset was split at the image level rather than the patient level, because patient/study identifiers were not retained in the annotation pipeline; we therefore cannot exclude the possibility that some patients contributed images to more than one split, which is a potential source of optimistic bias in the RSNA point estimates reported in Table 8. This risk does not apply to the BHSD experiment, which used a patient-level split by design (Section 3.2), and the consistency of the Attention U-Net’s advantage across both datasets (Section 5.1) should be read as partial, leakage-free corroboration of the RSNA finding rather than as removing this limitation. Second, both RSNA and BHSD use binary foreground masks without pixel-level hemorrhage subtype labels. Therefore, subtype-specific or small-lesion performance cannot be evaluated. Subtype-aware annotation and evaluation remain important directions for future work. Third, both models operate on individual 2D axial slices and do not exploit inter-slice 3D context. Although this reduces computational and annotation requirements, it may limit segmentation continuity across consecutive slices, particularly for hemorrhages extending across multiple slices. Fourth, although BHSD provides an independent test of cross-dataset reproducibility, evaluation on additional multi-center and publicly available datasets would be valuable to further assess the robustness of the proposed approach.

Future work will focus on leveraging unlabeled RSNA data through semi- or self-supervised learning, improving segmentation of imbalanced classes using combined loss functions, and exploring 3D or hybrid architectures to better capture spatial context with efficient computation. Given the BHSD findings, future work will also investigate dataset-specific calibration strategies (e.g., subtype-aware loss weighting, 2.5D or 3D context aggregation, and BHSD-specific hyperparameter tuning) aimed at narrowing the observed cross-dataset performance gap.

## 6. Conclusions

This study evaluated two models for binary ICH segmentation: a standard four-level U-Net and a proposed Attention U-Net with deep supervision, TTA, and cosine annealing, both trained on 1082 pixel-annotated RSNA images (of 20,154 total) annotated by experts using Roboflow, under identical splits, losses, batch sizes, and AMP settings.

The ablation study showed that each component contributed incrementally: cosine annealing (+0.0079 Dice; reduced instability), attention gates (+0.0048 Dice; reduced variability), deep supervision (+0.0251 sensitivity, crossing the 0.80 benchmarking reference value even without TTA), and TTA (+0.0034 Dice; +0.0053 IoU). These results support attention-guided, deeply supervised architectures with inference-time augmentation for ICH decision support, and motivate future semi-supervised use of RSNA’s unlabeled images.

To test generality, both architectures were retrained unchanged on the independent BHSD dataset (192 volumes; 2,961 slices). The Attention U-Net again outperformed the baseline on every metric (Dice 0.5676 vs. 0.5063; sensitivity 0.5350 vs. 0.5138; IoU 0.4408 vs. 0.3924)), confirming that the performance advantage of the proposed enhancements is maintained across the two evaluated datasets. This strengthens confidence that the observed advantage reflects a genuine architectural benefit rather than dataset-specific overfitting, although the possibility of patient overlap in the RSNA split (Section 5.2) means the RSNA point estimates alone should not be over-interpreted; the patient-level BHSD result is the more leakage-robust piece of evidence for this claim. Annotation granularity, lesion size, and patient diversity remain key determinants of absolute performance and a priority for future work.

As noted in Section 5.2, both models are binary, 2D, single-slice segmenters; subtype-resolved annotation and inter-slice 3D context remain open directions for follow-up work.

## Figures and Tables

**Table 1:** Training configuration for both architectures. All shared hyperparameters are held constant to ensure a controlled comparison. DS: deep supervision; TTA: test-time augmentation; AMP: automatic mixed-precision.

Hyperparameter	U-Net (Baseline)	Attention U-Net (Proposed)
Optimizer	Adam	Adam
Initial LR (η)	$1\times 10^{-4}$	$1\times 10^{-4}$
LR schedule	Fixed	Cosine annealing
$\eta_{\min}$	–	$1\times 10^{-6}$
$T_{\max}$	–	25
Loss function	Dice loss	DS Dice loss (Equation (5))
DS weights (λ)	–	1.0/0.4/0.3/0.2
Attention gates	No	Yes (all 4 levels)
$F_{\mathrm{int}}$	–	$F_{l}/2$
TTA at inference	No	Yes (orig + h-flip + v-flip)
Batch size	8	8
Epochs	25	25
AMP	Yes	Yes
Random seed	42	42

**Table 2:** Dataset distribution across training, validation, and test sets. Of the 20,154 total RSNA CT images, only 1082 carry pixel-level polygon annotations suitable for segmentation training.

Split	Samples	Percentage (%)	Purpose
Training	756	69.9	Model optimization
Validation	109	10.1	Hyperparameter tuning/checkpointing
Test	217	20.1	Final evaluation
Total (annotated)	1082	100.0	–
Total (full RSNA)	20,154	–	Unannotated images not used

**Table 3:** Baseline U-Net test set performance (217 samples, 25 epochs, batch size 8, 28 test batches, and AMP enabled).

Metric	Mean Score	Std Dev
Dice Similarity Coefficient	0.7683	±0.0906
Pixel Accuracy	0.9952	±0.0024
Precision	0.7901	±0.0841
Sensitivity (Recall)	0.7582	±0.1189
Specificity	0.9978	±0.0012
F1 Score	0.7683	±0.0906
IoU (Jaccard Index)	0.6322	±0.1143
Test Loss (Dice Loss)	0.3117	–

**Table 4:** Variant A—U-Net with cosine annealing LR only (no attention, no deep supervision, and no TTA). Test set: 217 samples.

Metric	Mean Score	Std Dev
Dice Similarity Coefficient	0.7762	±0.0910
Pixel Accuracy	0.9951	±0.0026
Precision	0.7791	±0.0846
Sensitivity (Recall)	0.7878	±0.1255
Specificity	0.9974	±0.0016
F1 Score	0.7762	±0.0910
IoU (Jaccard Index)	0.6427	±0.1147

**Table 5:** Variant B—Attention U-Net with cosine annealing LR (no deep supervision; no TTA). Test set: 217 samples.

Metric	Mean Score	Std Dev
Dice Similarity Coefficient	0.7810	±0.0803
Pixel Accuracy	0.9953	±0.0025
Precision	0.7858	±0.0800
Sensitivity (Recall)	0.7877	±0.1118
Specificity	0.9975	±0.0015
F1 Score	0.7810	±0.0803
IoU (Jaccard Index)	0.6474	±0.1018

**Table 6:** Variant C—Attention U-Net with cosine annealing LR and deep supervision (no TTA). Test set: 217 samples.

Metric	Mean Score	Std Dev
Dice Similarity Coefficient	0.7931	±0.0780
Pixel Accuracy	0.9954	±0.0024
Precision	0.7848	±0.0775
Sensitivity (Recall)	0.8128	±0.1080
Specificity	0.9974	±0.0015
F1 Score	0.7931	±0.0780
IoU (Jaccard Index)	0.6636	±0.0995

**Table 7:** Attention U-Net test set performance (217 samples, 25 epochs, AMP + cosine annealing + TTA enabled, batch size 8, and 28 test batches).

Metric	Mean Score	Std Dev
Dice Similarity Coefficient	0.7965	±0.0814
Pixel Accuracy	0.9955	±0.0024
Precision	0.7966	±0.0811
Sensitivity (Recall)	0.8094	±0.1145
Specificity	0.9976	±0.0014
F1 Score	0.7965	±0.0814
IoU (Jaccard Index)	0.6689	±0.1055
Test Loss (Dice Loss)	0.4425	–

**Table 8:** Comparative performance: baseline U-Net vs. proposed Attention U-Net on the test set (217 samples). Δ = Attention U-Net − U-Net. TTA applied to Attention U-Net at inference only.

Metric	U-Net	Attention U-Net	Δ	Better
Dice Score	0.7683	0.7965	+0.0282	Attention U-Net
Pixel Accuracy	0.9952	0.9955	+0.0003	Attention U-Net
Precision	0.7901	0.7966	+0.0065	Attention U-Net
Sensitivity	0.7582	0.8094	+0.0512	Attention U-Net
Specificity	0.9978	0.9976	−0.0002	U-Net
F1 Score	0.7683	0.7965	+0.0282	Attention U-Net
IoU (Jaccard)	0.6322	0.6689	+0.0367	Attention U-Net
Std Dev (Dice)	±0.0906	±0.0814	−0.0092	Attention U-Net
Std Dev (Sensitivity)	±0.1189	±0.1145	−0.0044	Attention U-Net
Std Dev (IoU)	±0.1143	±0.1055	−0.0088	Attention U-Net
Test Loss (Dice)	0.3117	0.4425	+0.1308	U-Net
Best Val Dice	0.7696	0.7833	+0.0137	Attention U-Net
Best Val Epoch	25/25	22/25	−3 epochs	Attention U-Net

**Table 9:** Ablation study summary: incremental effect of each component on test set Dice, Sensitivity, and IoU. Each row adds one component over the previous. A checkmark indicates that a component is included, while a multiplication sign indicates that it is not included. Sensitivity > 0.80 denotes crossing the 0.80 reference value used in this study for benchmarking purposes; this is not a clinically validated diagnostic threshold. TTA is applied at inference only.

Model Variant	Cosine LR	Attn Gates	Deep Sup.	TTA	Dice	Sensitivity	IoU	Best Val Dice
Baseline U-Net (fixed LR)	×	×	×	×	0.7683	0.7582	0.6322	0.7696
Variant A: + Cosine LR	✔	×	×	×	0.7762	0.7878	0.6427	0.7818
Variant B: + Attention Gates	✔	✔	×	×	0.7810	0.7877	0.6474	0.7868
Variant C: + Deep Supervision	✔	✔	✔	×	0.7931	0.8128	0.6636	0.7802
Proposed (Full Model)	✔	✔	✔	✔	0.7965	0.8094	0.6689	0.7833
*Total gain (Baseline → Proposed)*					+0.0282	+0.0512	+0.0367	

**Table 10:** Comparison of best validation and final test Dice scores for both models.

Model	Best Val Dice	Best Val Epoch	Test Dice	Δ (Test − Val)
U-Net	0.7696	25/25	0.7683	−0.0013
Attention U-Net	0.7833	22/25	0.7965	+0.0132

**Table 11:** BHSD dataset distribution across training, validation, and test sets. Splitting was performed at the patient level to prevent slice-level leakage.

Split	Patients	Slices	Percentage (%)	Purpose
Training	133	2035	68.7	Model optimization
Validation	20	305	10.3	Hyperparameter tuning/checkpointing
Test	39	621	21.0	Final evaluation
Total	192	2961	100.0	–

**Table 12:** Baseline U-Net test set performance on BHSD (621 samples, 25 epochs, batch size 8, AMP enabled, and fixed learning rate).

Metric	Mean Score	Std Dev
Dice Similarity Coefficient	0.5063	±0.2967
Pixel Accuracy	0.9971	±0.0027
Precision	0.5763	±0.2931
Sensitivity (Recall)	0.5138	±0.3299
Specificity	0.9986	±0.0016
F1 Score	0.5063	±0.2967
IoU (Jaccard Index)	0.3924	±0.2735
Test Loss (Dice Loss)	0.5147	–

**Table 13:** Attention U-Net test set performance on BHSD (621 samples, 25 epochs, AMP + cosine annealing + TTA enabled, and batch size 8).

Metric	Mean Score	Std Dev
Dice Similarity Coefficient	0.5676	±0.2963
Pixel Accuracy	0.9974	±0.0027
Precision	0.6654	±0.3043
Sensitivity (Recall)	0.5350	±0.3177
Specificity	0.9990	±0.0015
F1 Score	0.5676	±0.2963
IoU (Jaccard Index)	0.4408	±0.2801
Test Loss (Dice Loss)	0.4884	–

**Table 14:** Comparative performance on BHSD: baseline U-Net vs. proposed Attention U-Net on the test set (621 samples). Δ = Attention U-Net − U-Net. TTA applied to Attention U-Net at inference only.

Metric	U-Net	Attention U-Net	Δ	Better
Dice Score	0.5063	0.5676	+0.0613	Attention U-Net
Pixel Accuracy	0.9971	0.9974	+0.0003	Attention U-Net
Precision	0.5763	0.6654	+0.0891	Attention U-Net
Sensitivity	0.5138	0.5350	+0.0212	Attention U-Net
Specificity	0.9986	0.9990	+0.0004	Attention U-Net
F1 Score	0.5063	0.5676	+0.0613	Attention U-Net
IoU (Jaccard)	0.3924	0.4408	+0.0484	Attention U-Net
Std Dev (Dice)	±0.2967	±0.2963	−0.0004	Attention U-Net
Std Dev (Sensitivity)	±0.3299	±0.3177	−0.0122	Attention U-Net
Std Dev (IoU)	±0.2735	±0.2801	+0.0066	U-Net
Test Loss (Dice)	0.5147	0.4884	−0.0263	Attention U-Net
Best Val Dice	0.5226	0.5931	+0.0705	Attention U-Net

**Table 15:** Comparison with BHSD-based ICH segmentation methods. Dice scores are expressed as percentages for direct comparability with [26].

Method	Dim.	Supervision	Labeled Data	Dice (%)	Remarks
nnU-Net (SupOnly) [25]	3D	Full	96 volumes	45.10±0.21	Multi-class, binarized
Cross Pseudo Supervision + nnU-Net [26]	3D	Semi	96 labeled + 500 unlabeled	49.50±0.19	Best prior semi-supervised baseline
SSS (SAM-2 based) [26]	3D	Semi	96 labeled + 500 unlabeled	53.15±0.20	Previous state of the art on BHSD
Baseline U-Net (Ours)	2D	Full	133 patients (2035 slices)	50.63	Fixed LR, no attention
Attention U-Net (Ours)	2D	Full	133 patients (2035 slices)	56.76	Att. gates + DS + cosine LR + TTA

**Table 16:** Comparison with RSNA-based ICH segmentation methods.

Method	Dim.	Supervision	Dice	Remarks
Yüce et al. [22]	3D	Full	∼0.70	RSNA + QURE500
Ramananda & Sundaresan [23]	2D	Weak	0.583–0.640	RSNA
Song et al. [24]	3D	Full	∼0.79	nnU-Net
Baseline (Ours)	2D	Full	0.7683	U-Net
Proposed (Ours)	2D	Full	0.7965	Att U-Net (+0.0282 Dice)

## Data Availability

The RSNA Intracranial Hemorrhage Detection dataset is publicly available at: https://www.kaggle.com/c/rsna-intracranial-hemorrhage-detection (accessed on 12 December 2025). The Brain Hemorrhage Segmentation Dataset (BHSD) used for the secondary cross-dataset validation reported in this revision is publicly available as described in https://huggingface.co/datasets/Wendy-Fly/BHSD (accessed on 5 August 2026).

## Abbreviations

The following abbreviations are used in this manuscript:

ICH	Intracranial Hemorrhage
CT	Computed Tomography
IPH	Intraparenchymal Hemorrhage
SDH	Subdural Hematoma
EDH	Epidural Hematoma
IVH	Intraventricular Hemorrhage
SAH	Subarachnoid Hemorrhage
CAD	Computer-Aided Diagnosis
AI	Artificial Intelligence
RSNA	Radiological Society of North America
BHSD	Brain Hemorrhage Segmentation Dataset
COCO	Common Objects in Context
DSC	Dice Similarity Coefficient
IoU	Intersection over Union
AMP	Automatic Mixed Precision
AG	Attention Gate
TTA	Test-Time Augmentation
TP	True Positive
TN	True Negative
FP	False Positive
FN	False Negative
FCN	Fully Convolutional Network
AUC	Area Under the Curve
RNN	Recurrent Neural Network
CNN	Convolutional Neural Network
